# Winter conditions, not resource availability alone, may drive reversible seasonal skull size changes in moles

**DOI:** 10.1098/rsos.220652

**Published:** 2022-09-07

**Authors:** Lucie Nováková, Javier Lázaro, Marion Muturi, Christian Dullin, Dina K. N. Dechmann

**Affiliations:** ^1^ Department of Migration, Max Planck Institute of Animal Behavior, Am Obstberg 1, Radolfzell 78315, Germany; ^2^ Department of Zoology, Charles University, Viničná 7, Prague 128 00, Czech Republic; ^3^ Javier Lázaro Scientific and Wildlife Illustration, Gere Sopra 17, Noasca 10080, Italy; ^4^ Department for Diagnostic and Interventional Radiology, University Medical Center Goettingen, Robert-Koch-Straße 40, Goettingen 37075, Germany; ^5^ Department Translational Molecular Imaging, Max Planck Institute for Multidisciplinary Sciences, Herman-Rein-Straße 3, Goettingen 37075, Germany; ^6^ Department for Diagnostic and Interventional Radiology, University Hospital Heidelberg, Im Neuenheimer Feld 420, Heidelberg 69120, Germany; ^7^ Department of Biology, University of Konstanz, Universitätsstraße 10, 78464 Konstanz, Germany

**Keywords:** phenotypic flexibility, Dehnel's phenomenon, adaptation

## Abstract

Seasonal changes in the environment can lead to astonishing adaptations. A few small mammals with exceptionally high metabolisms have evolved a particularly extreme strategy: they shrink before winter and regrow in spring, including changes of greater than 20% in skull and brain size. Whether this process is an adaptation to seasonal climates, resource availability or both remains unclear. We show that European moles (*Talpa europaea*) also decrease skull size in winter. As resources for closely related Iberian moles (*Talpa occidentalis*) are lowest in summer, we predicted they should shift the timing of size changes. Instead, they do not change size at all. We conclude that in moles, seasonal decrease and regrowth of skull size is an adaptation to winter climate and not to a changing resource landscape alone. We not only describe this phenomenon in yet another taxon, but take an important step towards a better understanding of this enigmatic cycle.

## 1. Introduction

Organisms can change their morphology, behaviour or physiology during their lifetime to adapt to their environment, a phenomenon commonly summarized as phenotypic flexibility [1]. However, this process can be explained by different changes that animals can undergo in the course of their ontogeny (developmental plasticity), between generations (polyphenism), and/or as the result of changes in the environment (phenotypic plasticity). ‘Life cycle staging’ describes changes that are cyclical, reversible and linked to seasons, such as moulting, hibernation or migration, which happen within individuals. This kind of phenotypic variation is useful for studying adaptive processes because they are predictable and directly linked to environmental variables. Some traits, however, are typically exempt from such changes. For example, skeletal growth in mammals typically slows down drastically or stops altogether once individuals reach adulthood, aside from some bone degeneration in very old individuals. A poignant exception to this occurs in a handful of small mammals with exceptionally high metabolisms, high-quality diets, that remain active year-round. These animals, in a reversible process called Dehnel's phenomenon, shrink their size by 20% or more in anticipation of winter, including many organs and tissues [2,3].

Regrowth occurs in the spring, with some organs exceeding the first summer size, while others regrow only partially [4]. However, the most remarkable feature of this phenomenon is the massive change in skull size and brain mass, which entails a great capacity for flexibility in brain tissue and ossified bone. This phenomenon, discovered in 1949 [5], received little attention until recently, when the applied relevance of such massive tissue regeneration involved in the process was discovered. It is by far best described in red-toothed shrews [6], particularly in the common shrew, *Sorex araneus* Linnaeus, 1758, but has recently also been described in two species of small mustelids [7]. This not only emphasizes that Dehnel's phenomenon may be a successful wintering strategy across taxa, but also allows broader hypothesis testing in regard to the evolutionary selective pressures that have led to these seasonal size changes. While the spring regrowth is thought to be in preparation for (often/usually terminal) reproduction, physiological studies of the common shrew show that in spite of one of the highest measured mammalian metabolisms, shrews' relative energy use, i.e. oxygen consumption per unit of body mass, does not change between seasons despite a change of ambient temperatures of at least 30°C [8]. Thus, a body mass decrease of 20% provides absolute energy savings that may enable the animals’ survival in winter. These savings might be enhanced by decreasing energetically expensive tissue such as the brain. However, it remains unclear whether Dehnel's phenomenon is a response to reduced resource availability, harsher climatic conditions, or both. This uncertainty is enhanced by the fact that the main model organism to study Dehnel's phenomenon, the common shrew, has a mean lifespan of only 13 months and thus cycles through the phenomenon only once.

**Figure 1.**
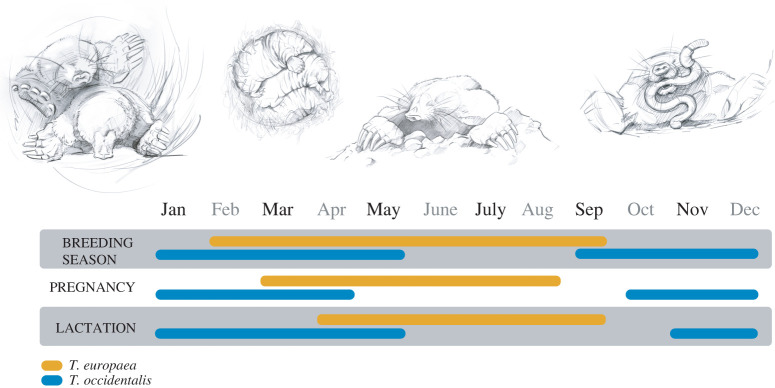
Life cycle of European mole (*T. europaea*, yellow lines) and Iberian mole (*T. occidentalis*, blue lines) indicating entire breeding season (reproductive period), pregnancy and lactation, based on [11].

We have identified a taxon, moles, where the alternative hypotheses: (i) Dehnel's phenomenon is a response to lower seasonal resource availability, (ii) Dehnel's phenomenon is a response to harsher/colder climates in winter, or (iii) both can be addressed. Our preliminary study of specimens in the Zoological collection of the Mammal Research Institute, Polish Academy of Sciences (Poland, electronic supplementary material, figure S1) and notes in one study from the Czech Republic [9] indicated that the European mole (*Talpa europaea* Linnaeus, 1758), a species phylogenetically closely related to *Sorex*, may also exhibit Dehnel's phenomenon and decrease relative skull size in winter. Moles, similar to shrews, are small mammals with a high energy expenditure, especially when digging, that do not hibernate and have limited possibility to migrate. Two closely related mole species [10] occur in regions with very different climatic regimes, thus allowing testing hypotheses about whether or not they should exhibit Dehnel's phenomenon and when. The Iberian mole (*Talpa occidentalis* Cabrera, 1907), with a distribution restricted to Portugal and Spain, has a seasonally shifted reproductive cycle compared with the European mole (figure 1), with pregnancy and lactation occurring in the milder winter months, while European moles reproduce in spring and summer. We thus hypothesized that (i) the European mole exhibits Dehnel's phenomenon and that the temporal pattern of size change would match the pattern found in shrews and mustelids with relative skull size becoming smaller in winter and regrowing in spring; (ii) the Iberian mole either also exhibits Dehnel's phenomenon, but with the smaller relative size occurring in summer, when resources are low and conditions are harsher compared with winter, or (iii) the Iberian mole would not exhibit Dehnel's phenomenon. In the latter case, this would indicate that Dehnel's phenomenon is an adaptation that is at least not only caused by low resource availability but may also be tied to winter climate. We thus aimed to confirm that Dehnel's phenomenon occurs in the genus *Talpa* and to test these hypotheses by comparing skull measurements from year-round samples in both mole species by especially comparing summer individuals with winter individuals of the Iberian mole, and summer individuals with winter individuals of the European mole.

## 2. Material and methods

### 2.1. Skull measurements

Skulls of both species were measured using the methods described for shrews [3] and weasels [7]. We measured skull length (SKL), braincase height (SKH), braincase width (SKB), length of the mandible (ML) and length of the molar tooth row (MTR, electronic supplementary material, figure S2). Skull length was measured from the most anterior point of the maxilla to the occipital condyle, braincase height from the tympanic rings to the dorsal part of the skull and braincase width as the maximum width of the braincase. Length of the mandible was taken from the alveolus dentalis of the first incisor to the most posterior part of the coronoid process. Length of the molar tooth row was taken from the most anterior part of the first lower molar alveolus to the most posterior part of the dental *alveolus* of the third lower molar. All measurements were taken by digital calliper with a precision of 0.01 mm.

A preliminary dataset of European moles from the collection at the Mammal Research Institute in Białowieza (Poland) had indicated that moles might exhibit Dehnel's phenomenon (electronic supplementary material, figure S1). As we wanted to test if European moles exhibit Dehnel's phenomenon and because there could be geographical variation in phenology as in *Sorex araneus* [6], we selected individuals from the whole life cycle stored in the collections of the Institute of Vertebrate Biology at the Czech Academy of Sciences in Brno. All skulls were measured by a single observer (L.N.). To minimize other effects, we chose specimens from the same region, Moravia, in the Czech Republic. We aged skulls based on tooth wear in combination with collection date to distinguish first-year animals from older ones. We additionally classified individuals based on their life cycle beginning on 1 May into age classes as follows [11]: young individuals with little tooth wear in the first year of their life, mature individuals with medium tooth wear and finally old individuals with strong tooth wear. Unfortunately, we did not have any specimens from February.

We measured skulls of *T. occidentalis* from the collection of the Estación Biológica de Doñana in Spain. All skulls of *T. occidentalis* were measured by a single observer (M.M.). The measurements were taken the same way as in *T. europaea*. Classification to the age group was based on tooth wear as in *T. europaea*, but the beginning of the life cycle based on the Iberian mole's reproductive cycle was 1 January [11]. As we were testing alternative hypotheses (resource availability versus winter climate), we measured skulls from the entire year when available and matched them to corresponding months in the European mole life cycle as well as local life-history stages of the Iberian mole (figure 1). See electronic supplementary materials (electronic supplementary material, table S1) for a complete list of specimens and measurements. Measurement error was determined by both observers (L.N. and M.M.) measuring the same subset of skulls (electronic supplementary material, table S2).

### 2.2. CT scans and skull shape analysis

To describe changes in external size, shape and bone thickness, we obtained high-resolution CT scans from exemplary skulls of both species using the *in vivo* microCT ‘QuantumFX’ (Perkin Elmer) operated with the following settings: tube voltage 90 kV, tube current 200 µA, field of view 20 × 20 × 20 mm^3^ resulting in three-dimensional datasets with a pixel size of 40 µm. To cover the entire skull, two scans were performed and combined using a custom-made stitching algorithm. From these datasets, surface models were extracted using VGStudioMax v. 3.1 (VolumeGraphics), simplified and scaled according to the differences in the raw length of the jaw in Meshlab [12]. To display the local seasonal deformation, the Housedorff difference was calculated and colour coded (Meshlab).

### 2.3. Statistical analyses

As described in other species, moles may undergo size-specific selection in autumn with larger individuals disappearing from the population. This can cause an average size decrease from summer to winter at the population level, which could mask the individual skull changes that we wanted to discover. To account for the potential size-selection effect and size differences caused by sexual dimorphism [9,13], we standardized our braincase measurements by using the ratio of braincase height and length of the molar tooth row, which remains constant along the year.

As we expected to find seasonal change in relative skull size similar to the patterns found in shrews [3,14], we tested for nonlinearity using generalized additive models (GAMs) with Gaussian distribution. As a non-parametric term, we used the ‘day of life’ calculated as the Julian day of their capture date with ‘one’ set to 1^st^ May for *T. europaea* and 1^st^ January for *T. occidentalis* as an approximation of their dates of birth [11]. When we only had the capture month, we used the 15th of the corresponding month. To this modified Julian day, we added years according to their estimated age as described above. We included sex as a parametric term and applied a smoothing function to the ‘day of life’ with *k* = 7, based on previous models used for shrews [3,14], where a similar pattern was fitted with *k* = 5 but accounting for the longer lifspan and of moles and the expected possibility of a second cycle of size change. We kept these parameters for the GAMs in both species, so we could test for significance in nonlinearity and describe the potential seasonal patterns in each species.

We additionally tested for differences of *T. europaea* relative braincase height between all combinations of all months to reveal the pair of months with the most pronounced differences using a pairwise *t*-test. We did not have enough data to test each age group separately and pooled all age groups for this test. As we tested several pairs of variables, we corrected our *p*-values for multiple testing with the Holm–Bonferroni method. Results for all combinations of months are shown in the electronic supplementary material, table S3. All analyses were performed in R v. 4.0.5 [15].

## 3. Results

### 3.1. Talpa europaea

**Figure 2.**
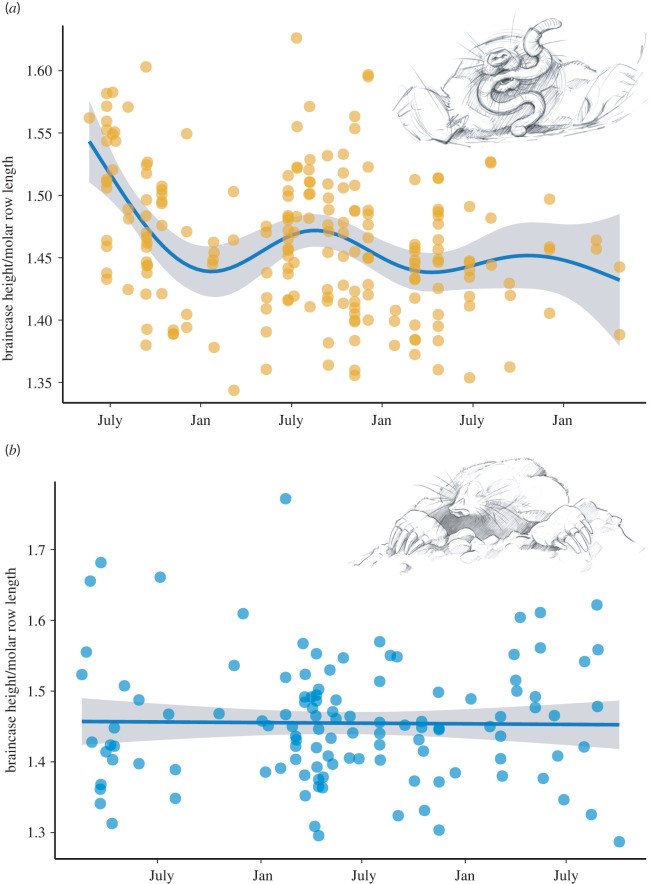
Variation in relative braincase height over the lifespan of *T. europaea* (*a*) and *T. occidentalis* (*b*). Lines and shadowed areas represent fitted GAMs and 95% confidence intervals respectively. See text for details.

**Figure 3.**
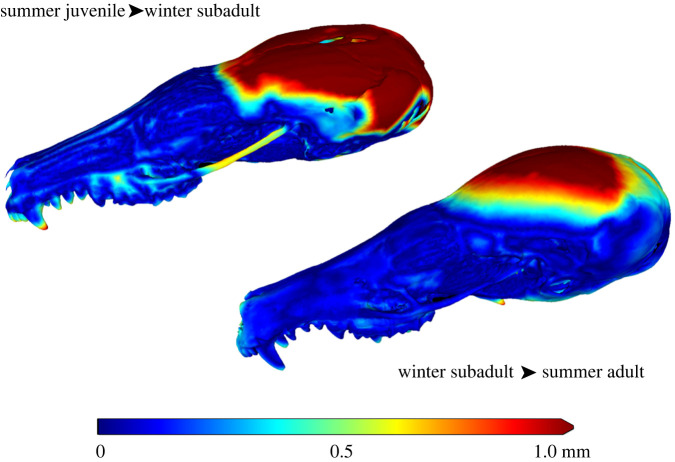
Local seasonal deformation of the skull in *T. europaea*. The absolute distance of an exemplary winter skull of a subadult individual to a summer juvenile skull (not shown) and between the skull of a summer adult and the skull of a winter subadult is shown. Note: the surface models were scaled according to the molar row length to remove the influence of the growth. Clearly, a strong reduction and a later remodelling of the height of the brain case is visible.

We found evidence for reversible skull size changes in the European mole (figure 2). In *T. europaea* relative skull height followed a seasonal pattern along their lifespan, with maxima in summer and minima in winter (figures 2 and 3). These size changes were confirmed by our model as a significant nonlinear pattern (GAM, *n* = 202, e.d.f. (smooth term) = 5.13, *p* < 0.001). Freshly dispersing juvenile moles had the highest absolute and relative skull height. Individuals then shrank their relative skull size by 11%, reaching minimum size in November of the first year of their life. In the following spring, skull size increased again with a second, lesser peak occurring by July (regrowth by 4%). This was followed by another decline and potential regrowth; however, our sample size became smaller with increasing age and was too small to test for significant differences in older individuals. We used a *t*-test to also confirm significant differences in relative braincase height between winter and summer season (electronic supplementary material, table S3).

### 3.2. Talpa occidentalis

This species is overall smaller than *T. europaea*, which is reflected in absolute skull size (electronic supplementary material, figure S3). Unlike *T. europaea,* relative skull height did not change significantly (GAM, *n* = 104, e.d.f. (smooth term) < 0.001, *p* > 0.5; figure 2). There was no significant size change either during the harshest time of the year in terms of resources (the local summer), or at the ages when *T. europaea* changes size.

## 4. Discussion

Our analyses of large skull collections support the hypothesis that the European mole, *T. europaea*, exhibits Dehnel's phenomenon, which was only previously described in red-toothed shrews and small mustelids [6]. Similar to mustelids and shrews, we found a significantly lower relative braincase height and thus presumably brain size in winter than in the first summer and following spring of *T. europaea*'s life. However, we did not find Dehnel's phenomenon in the closely related Iberian mole, *T. occidentalis*. This has important implications for the understanding of this remarkable phenomenon. It leads to the conclusion that Dehnel's phenomenon is an adaptation that is tied to winter climate and at least not only caused by low resource availability.

The species, which currently have been described to exhibit Dehnel's phenomenon share several common traits such as year-round activity, a high metabolism linked to high-quality diet and a low ability to store fat or enter energy-saving states such as torpor or hibernation [6,16]. In addition, the species that have Dehnel's phenomenon have their core distributions at high latitudes with harsh conditions and low temperatures in the winter. By decreasing mass in winter, especially of energetically expensive tissues such as the brain, these animals probably reduce their overall energy consumption and thus the amount of food needed [17–19]. The prevailing hypotheses were thus that Dehnel's phenomenon is an adaptation to harsh climates in winter, a decrease in resource availability, or both. The contrasting results of the two mole species in this study give support to the winter adaptation hypothesis. Although the Iberian mole seasonally faces very harsh conditions, these conditions occur in summer, when it is extremely hot and dry in its habitat [20,21]. We predicted that low resource availability during this season might cause the Iberian mole to decrease size in summer, similar to a shift in hibernation strategy in hedgehogs [22] where some temperate species can hibernate (*Erinaceus* sp.) and others, occurring in warm and dry climates, aestivate (*Hemiechinus* sp.), *sensu* [1]. The fact that the Iberian mole does not decrease relative skull size either in summer or in winter thus supports the hypothesis that winter conditions are at least partly responsible for the evolution and/or occurrence of Dehnel's phenomenon. Dehnel's phenomenon is also not simply linked to a certain developmental stage or age (figure 2). Due to the low number of moles with high tooth abrasion (i.e. old animals) in our dataset we could not support our statement with a rigid test; however, the shrinking and regrowth occur repeatedly in *T. europaea* (figure 2). As European moles are born throughout spring, the age of animals in winter may differ by several months, again supporting that Dehnel's phenomenon is linked to the changing environment rather than the developmental stage of an individual.

Thus, the evolution of Dehnel's phenomenon remains unresolved but intriguing. The presence of reversible seasonal skull size changes in the genus *Talpa* provides important clues but also raises questions if the evolution of this phenomenon is ancestral or convergent or a mixture of both. All species of Eurasian red-toothed shrews (subfamily Soricinae) investigated to date show the phenomenon [6,23]. Interestingly, there are alternative postulated topologies within the Eulipotyphla clade, where the moles (family Talpidae) and shrews (Soricidae) are included in the monophyletic group Soricomorpha [24]. In this scenario, Dehnel's phenomenon may have evolved at the root of the group in the common ancestor of all Soricomorpha. Warm-adapted clades such as the white-toothed shrews (subfamily Crocidurinae) and *T. occidentalis* would then have secondarily lost Dehnel's phenomenon similar to the loss of echolocation in some bats [25]. In the second, alternative topology, moles (Talpidae) are a separate monophyletic group, and importantly, the hedgehogs and gymnures (Erinaceidae) are a sister group of the shrews [26,27]. In this scenario, the moles, like the weasels, would have evolved Dehnel's phenomenon independently. Testing the differently placed hedgehogs, depending on the topology, for the presence of Dehnel's phenomenon might be interesting here. However, hedgehogs have an entirely different wintering/low resource availability strategy from the high-paced shrews, moles and mustelids. They store fat and are able to hibernate (or aestivate in the case of *Hemiechinus*) and would not be predicted to show Dehnel's phenomenon.

Further study will be necessary to understand the evolutionary pattern(s) of this phenomenon and infer if it evolved several times, as well as to tease apart the roles of different evolutionary drivers. Regardless of the phylogeny of the Eulipotyphla, the fact that weasels and stoats (*Mustela* spp., order Carnivora) also exhibit Dehnel's phenomenon indicates convergent evolution of the phenomenon in several taxa. Geographical analysis of Dehnel's phenomenon in red-toothed shrews indicates that skull size changes are stronger at higher latitudes, where winter conditions are harsher [6]. Weasels have a distribution range that extends into very arid regions [28], potentially allowing the comparison of populations that have lived under very different conditions for long time periods within the same species. Similarly, all red-toothed shrews studied to date show Dehnel's phenomenon, but only one of the two mole species we studied have Dehnel's phenomenon. The study of additional mole species might thus also be useful as there is already cursory mention of skull size changes in another species occurring partially also in cold habitats, the blind mole *T. caeca* [29]. However, the study of Dehnel's phenomenon is difficult, as skull collections are nearly always seasonally biased and field studies require recapture and X-raying [3] or sacrificing a large number of animals across at least one entire year. Yet, only the study of more species, such as temperate hedgehogs, and the reconstruction of the ancestral traits in complete or largely complete phylogenetic trees will allow us to fully tease apart the conflicting constraints of ancestry and environmental pressure during evolution.

## Data Availability

The datasets supporting this article have been uploaded as part of the electronic supplementary material [30].
